# Nationwide population-based study of prevalence and trend of borderline ovarian tumors in the Republic of Korea

**DOI:** 10.1038/s41598-021-90757-8

**Published:** 2021-05-27

**Authors:** Yung-Taek Ouh, Dongwoo Kang, Hoseob Kim, Jae Kwan Lee, Jin Hwa Hong

**Affiliations:** 1 Department of Obstetrics and Gynecology, School of Medicine, Kangwon National University, Kangwon, Korea; 2 Data Science Team, Hanmi Pharmaceutical. Co. Ltd, Seoul, Korea; 3 Department of Obstetrics and Gynecology, Guro Hospital, College of Medicine, Korea University, 148 Gurodong-ro, Guro-gu, Seoul, 08308 Korea

**Keywords:** Cancer epidemiology, Gynaecological cancer

## Abstract

Borderline ovarian tumors (BOTs) represent noninvasive tumors with uncertain malignant potential. They have a favorable prognosis although they can also recur or be fatal. There are limited population-based data on BOTs, its incidence and surgical treatment approach. We sought to analyze these trends in South Korea between 2014 and 2018. Data from patients diagnosed with BOT between 2014 and 2018 were obtained from the Health Insurance Review and Assessment Service/National Inpatient Sample in South Korea. Treatment was analyzed by using codes including adnexal surgery with or without hysterectomy. Data from 4,636,542 women were entered into the database between 2014 and 2018. Data from 5,109 women with BOT, and 537 women with surgery were extracted for analysis. The highest prevalence of BOT occurred in women 40–44 years old. In logistic regression analysis, age was significantly correlated with the prevalence of BOT (p < 0.05). The prevalence of BOT was lower in individuals over 50 than it was in those under 50 years (odds ratio (OR), 0.400 in 2014; OR, 0.457 in 2015; OR, 0.419 in 2016; OR, 0.355 in 2017; OR, 0.347 in 2018). The prevalence of BOT varies significantly with age, and is most common in women in their 40 s.

## Introduction

Unlike invasive ovarian cancer, borderline ovarian tumor (BOT) mostly occurs in young women and is often diagnosed at an early stage. Therefore, most cases of BOT have an excellent prognosis with > 95% of overall survival. BOT is characterized by a certain extent of cellular proliferation and nuclear atypia in the absence of infiltrative growth and/or apparent stromal invasion. BOT accounts for up to 20% of all epithelial ovarian tumors, and is classified as a separate entity from benign ovarian tumor and epithelial ovarian carcinoma^1^.

The exact incidence of BOT is still unknown. According to SEER (surveillance epidemiology and end results) data, there are 2.5 new cases per 100,000 women-years in the USA^2^. The incidence rates of BOT have increased in recent decades, while the incidence rates of invasive ovarian cancer has been decreased in Western countries^3^. The most recent study of BOT incidence from Denmark showed that the incidence of serous BOT has stabilized, while that of mucinous BOT has decreased^4^. BOT usually occurs in relatively young women and has good prognosis, even if there are metastatic lesions in the peritoneal cavity^5^. The 5-year survival rate of stage I–III BOT is approximately 98%^4^. Nevertheless, some patients developed BOT recurrence, especially with invasive carcinoma.

Standard surgical treatment for BOT is recommended for systemic surgical staging. This staging includes multiple peritoneal biopsies, omentectomy, bilateral salpingo-oophorectomy and hysterectomy. The majority of patients with BOT is diagnosed at an early stage. These tumors are often diagnosed incidentally during surgery. Therefore, the surgical management of BOT is heterogeneous. The population-based incidence or management of BOT has only been reported in some European countries, and have not been investigated in many countries. Epidemiologic study provide some risk factor differences across racial/ethnic groups that may lead to prevent and improve survival across all racial/ethnic groups. Therefore, more recent and domestic data are needed to clarify the actual incidence rates and management of BOT. We investigated the longitudinal incidence rates of BOT in South Korea between 2014 and 2018 using population-based nationwide health insurance claims data. Analyzing the recent trends of BOT by age might provide evidence for the diagnosis and management of patients with BOT.

## Results

**Figure 1:**
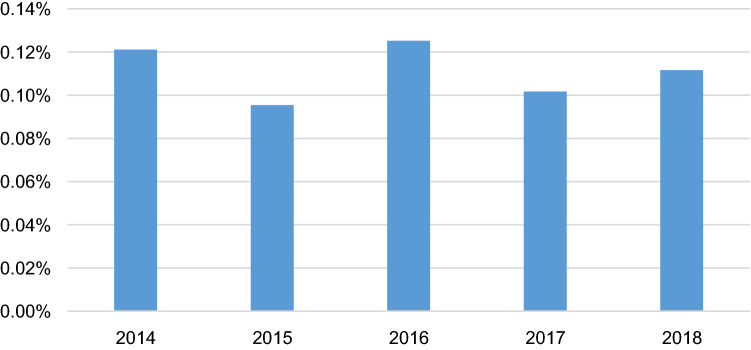
The annual trends in the prevalence of borderline ovarian tumors from 2014 to 2018.

Of a total of 4,636,542 women, 5,109 met the inclusion criteria with a diagnosis of BOT (Table 1). The prevalence of BOT was 1,033 in 2014 (0.12%), 1,235(0.10%) in 2015, 1,237 (0.13%) in 2016, 757 (0.10%) in 2017, and 847 (0.11%) in 2018. These prevalence rates differed significantly, but did not show a trend (Fig. 1). The median age of patients with BOT was 44.56 (± 15.45) years in 2014, 43.90 (± 15.36) in 2015 and 45.26 (± 15.69) in 2016. The BOT was most prevalent in women aged 45 to 49 in both 2017 (14.27%) and 2018 (12.99%) (Supplementary Table 1). The primary treatment was surgical in 92 patients (12.15%) in 2017, followed by 123 (11.91%) in 2014, 123 (9.94%) in 2016, 119 (9.64%) in 2015, and 80 (9.45%) in 2018, respectively. There were no significant differences in the proportion of surgical procedures between 2014 and 2018. The prevalence of the surgical approach used, including laparotomy, conventional laparoscopy, and single-port laparoscopy, did not differ during the study period. The proportion of laparotomy and laparoscopy was not significantly different by year (between 2014 and 2018). In contrast, the type of surgery whether it spared fertility or not differed year by year during the study period without any significant increasing or decreasing trend (Table 2).

**Table 1:** Characteristics associated with borderline ovarian tumors from 2014 to 2018.

	2014	2015		2016	2017	2018	p-value
Number of patients	1,033 (0.12%)		1,235 (0.10%)	1,237 (0.13%)	757 (0.10%)	847 (0.11%)	< 0.0001
Age, mean ± SD	44.56 ± 15.45		43.90 ± 15.36	45.26 ± 15.69	N/A	N/A	
**Surgery**							
No	910 (88.09%)		1,116 (90.36%)	1,114 (90.06%)	665 (87.85%)	767 (90.55%)	
Yes	123 (11.91%)		119 (9.64%)	123 (9.94%)	92 (12.15%)	80 (9.45%)	0.0072
SO/cystectomy	89 (8.62%)		79 (6.40%)	90 (7.28%)	70 (9.25%)	56 (6.61%)	0.005
TH + SO/cystectomy	14 (1.36%)		13 (1.05%)	10 (0.81%)	9 (1.19%)	8 (0.94%)	0.6919
SO/cystectomy + Surgical staging	22 (2.13%)		30 (2.43%)	26 (2.10%)	15 (1.98%)	14 (1.65%)	0.7904
Ovarian wedge resection	1		2	1	4 (0.53%)	2	0.3021
**Surgical approach**							
Laparotomy	56 (5.42%)		45 (3.64%)	55 (4.45%)	39 (5.15%)	34 (4.01%)	0.9077
**Laparoscopy**							
Single-port	4 (0.39%)		11 (0.89%)	10 (0.81%)	3 (0.40%)	6 (0.71%)	0.6820
Conventional	63 (6.10%)		63 (5.10%)	58 (4.69%)	50 (6.61%)	40 (4.72%)	0.9323

*TH* Total hysterectomy, *SO* salpingo-oophorectomy, *N/A* not applicable: In 2017 and 2018, only frequency by age group was provided, not by age of individual patients.

**Table 2:** Annual trends in surgical approach and fertility-sparing surgery.

Years	2014	2015	2016	2017	2018	P value
	No	No	No	No	No	
**Surgical approach**						
Laparotomy	67 (54.5%)	74 (62.2%)	68 (55.3%)	53 (57.6%)	46 (57.5%)	0.7776
Laparoscopy	56 (45.5%)	45 (37.8%)	55 (44.7%)	39 (42.4%)	34 (42.5%)	
**Fertility-sparing**						
Yes	98 (77.8%)	93 (75.0%)	101 (79.5%)	80 (81.6%)	61 (76.3%)	0.039
No	28 (22.2%)	31 (25.0%)	26 (20.5%)	18 (18.4%)	19 (23.7%)	

**Figure 2:**
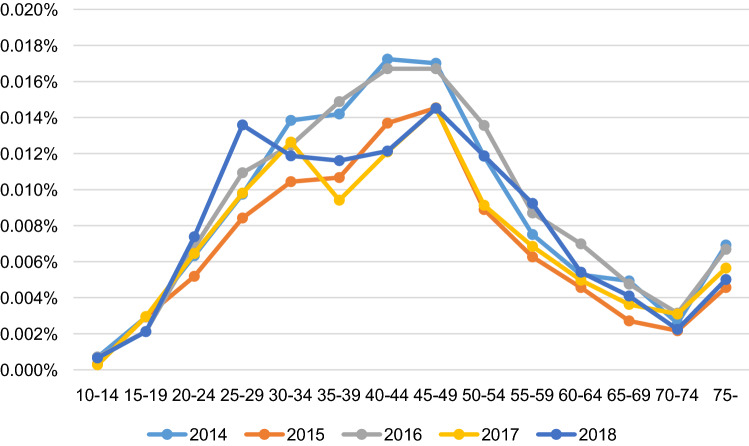
The serial trends in the prevalence of BOT from 2014 to 2018 stratified according to 5-year age increments.

The age-specific prevalence rates of BOT in the study period were described in Table 3, in which the annual prevalence was not significantly different (p = 0.4454). According to logistic regression analysis, age significantly correlated with BOT prevalence (p < 0.05) (Table 4). The most prevalent age of borderline ovarian tumors was between 40 and 44 years of age, and the prevalence of borderline ovarian tumors gradually decreased with increasing age. Compared to women aged 10 to 14 years old, the prevalence of BOT was higher in all women under the age of 69 from 2014 to 2016. There was only a significant difference in those under 59 years of age in 2017 and 2018. In addition, there was no significant difference in the annual prevalence of BOT from 2016 to 2018 compared to 2014. The prevalence rate was higher among women with low socioeconomic level compared with women with higher levels in 2014 (OR, 1.397; p < 0.05). However, the prevalence rate of BOT did not differ according to socioeconomic status from 2015 to 2018. The highest prevalence of BOT occurred in patients between 40 and 44 years old. In contrast, the lowest prevalence of BOT occurred in patients under 20 years old. Almost all age groups had a higher prevalence rate of BOT than did the 10–14 year old age group. However, the prevalence rate of women over 70 years old was not significantly different from those aged 10–14 years. The trends in the BOT prevalence rates from 2014 to 2018 stratified according to 5-year age increments are illustrated in Fig. 2.

**Table 3:** Age-specific prevalence rates of borderline ovarian tumors from 2014 to 2018 stratified according to 5-year age increments.

Age (years)	2014	2015		2016	2017	2018	P value
	No	No		No	No	No	
0–14	5 (0.03%)	4 (0.01%)		7 (0.03%)	2 (0.01%)	5 (0.04%)	0.4454
15–19	23 (0.09%)	33 (0.06%)		21 (0.07%)	22 (0.11%)	16 (0.08%)	
20–24	51 (0.17%)	67 (0.11%)		61 (0.16%)	48 (0.18%)	56 (0.21%)	
25–29	84 (0.20%)	108 (0.16%)		109 (0.24%)	73 (0.22%)	103 (0.31%)	
30–34	116 (0.17%)	135 (0.14%)		118 (0.17%)	94 (0.21%)	90 (0.21%)	
35–39	112 (0.23%)	134 (0.16%)		153 (0.26%)	70 (0.16%)	88 (0.20%)	
40–44	151 (0.28%)	172 (0.19%)		154 (0.27%)	90 (0.21%)	92 (0.22%)	
45–49	145 (0.23%)	190 (0.19%)		174 (0.25%)	108 (0.20%)	110 (0.21%)	
50–54	110 (0.14%)	119 (0.11%)		134 (0.17%)	68 (0.11%)	90 (0.15%)	
55–59	63 (0.09%)	87 (0.08%)		85 (0.10%)	51 (0.07%)	70 (0.10%)	
60–64	48 (0.09%)	60 (0.07%)		75 (0.11%)	37 (0.06%)	41 (0.06%)	
65–69	40 (0.08%)	36 (0.05%)		44 (0.07%)	27 (0.05%)	31 (0.06%)	
70–74	23 (0.04%)	27 (0.04%)		30 (0.05%)	23 (0.05%)	17 (0.04%)	
75-	61 (0.06%)	61 (0.05%)		71 (0.06%)	42 (0.04%)	38 (0.03%)	
Total	1,032 (0.12%)	1,233 (0.10%)		1,236 (0.13%)	755 (0.10%)	847 (0.11%)

**Table 4:** Multivariate logistic regression analysis of the risk factors of borderline ovarian tumors.

	2014		2015		2016		2017		2018	
	OR (95% CI)	P value	OR (95% CI)	P value	OR (95% CI)	P value	OR (95% CI)	P value	OR (95% CI)	P value
		Ref	0.788 (0.725–0.856)	< .001	1.052 (0.968–1.143)	0.231	0.844 (0.768–0.927)	< .001	0.930 (0.849–1.018)	0.115
5-year patient age range										
10–14		Ref		Ref		Ref		Ref		Ref
15–19	3.130 (1.190–8.233)	< 0.05	6.101 (2.161–17.221)	< 0.001	2.091 (0.889–4.918)	0.091	2.255 (0.826–6.156)	0.113	2.255 (0.826–6.156)	0.113
20–24	5.731 (2.287–14.36)	< 0.001	11.13 (4.059–30.523)	< 0.001	4.906 (2.244–10.726)	< 0.001	6.003 (2.404–14.988)	0.0001	6.003 (2.404–14.988)	< 0.001
25–29	7.221 (2.929–17.803)	< 0.001	16.357 (6.029–44.372)	< 0.001	7.315 (3.406–15.711)	< 0.001	8.923 (3.636–21.899)	< .0001	8.923 (3.636–21.899)	< 0.001
30–34	5.881 (2.402–14.397)	< 0.001	14.17 (5.242–38.302)	< 0.001	5.342 (2.492–11.452)	< 0.001	6.094 (2.476–14.999)	< .0001	6.094 (2.476–14.999)	< 0.001
35–39	7.321 (2.989–17.934)	< 0.001	15.524 (5.742–41.968)	< 0.001	7.354 (3.447–15.688)	< 0.001	5.624 (2.284–13.85)	0.0002	5.624 (2.284–13.85)	< 0.001
40–44	9.617 (3.945–23.442)	< 0.001	19.341 (7.177–52.12)	< 0.001	8.094 (3.795–17.266)	< 0.001	6.383 (2.595–15.704)	< .0001	6.383 (2.595–15.704)	< 0.001
45–49	8.133 (3.334–19.835)	< 0.001	19.533 (7.256–52.581)	< 0.001	7.592 (3.566–16.163)	< 0.001	5.952 (2.429–14.587)	< .0001	5.952 (2.429–14.587)	< 0.001
50–54	4.971 (2.028–12.18)	< 0.001	10.911 (4.029–29.55)	< 0.001	5.115 (2.392–10.938)	< 0.001	4.355 (1.769–10.718)	< 0.01	4.355 (1.769–10.718)	< 0.01
55–59	2.877 (1.157–7.151)	0.023	7.788 (2.859–21.218)	< 0.001	2.791 (1.291–6.033)	< 0.01	2.748 (1.109–6.811)	< 0.05	2.748 (1.109–6.811)	0.029
60–64	2.818 (1.122–7.08)	0.028	6.886 (2.503–18.946)	< 0.001	2.946 (1.358–6.393)	< 0.01	1.84 (0.727–4.657)	0.198	1.84 (0.727–4.657)	0.198
65–69	2.534 (1.000–6.422)	0.05	4.853 (1.727–13.634)	< 0.05	2.072 (0.933–4.601)	0.073	1.682 (0.654–4.327)	0.281	1.682 (0.654–4.327)	0.281
70–74	1.366 (0.519–3.594)	0.527	3.797 (1.329–10.852)	0.0128	1.48 (0.65–3.369)	0.351	1.045 (0.385–2.832)	0.931	1.045 (0.385–2.832)	0.931
75-	1.816 (0.73–4.521)	0.200	4.293 (1.561–11.806)	0.0048	1.541 (0.709–3.349)	0.275	0.977 (0.384–2.482)	0.961	0.977 (0.384–2.482)	0.961
SES										
High		Ref		Ref		Ref		Ref		Ref
Low	1.397 (1.042–1.872)	< 0.05	0.88 (0.637–1.216)	0.438	1.09 (0.814–1.459)	0.564	1.085 (0.744–1.583)	0.671	0.792 (0.523–1.199)	0.271

*OR* odds ratio, *SES* socioeconomic status, *OR* odds ratio, *CI* confidence interval.

The age-standardized prevalence rates stratified are shown in Table 5. Compared to women in their 40 s, the prevalence of BOT was significantly less prevalent in patients 10–19 years old (OR, 0.257; p < 0.001 in 2014; OR, 0.202; p < 0.001 in 2015; OR, 0.210; p < 0.001 in 2016; OR, 0.332; p < 0.001 in 2017; OR, 0.283; p < 0.001 in 2018). Furthermore, BOT was less prevalent in patients in their 60 s compared to that in other age groups (p < 0.001). The prevalence of BOT was not significantly different between patients 10–19 years old and those over 60 years between 2014 and 2018. Consistently throughout the study period, the prevalence of BOT was less prevalent in those over age 50 than it was in those under 50 years (OR, 0.400; p < 0.001 in 2014; OR, 0.457; p < 0.001 in 2015; OR, 0.419; p < 0.001 in 2016; OR, 0.355; p < 0.001 in 2017; OR, 0.347; p < 0.001 in 2018).

**Table 5:** Age-standardized prevalence rate stratified by age from 2014 to 2018.

	2014			2015			2016				2017			2018		
Age	No	OR	p-value	No	OR	p-value	No		OR	p-value	No	OR	p-value	No	OR	p-value
40–49	296 (0.256%)	Ref		362 (0.192%)	Ref		328 (0.259%)		Ref		198 (0.209%)	Ref		202 (0.215%)	Ref	
10–19	28 (0.066%)	0.257	< 0.001	37 (0.040%)	0.202	< .0001	28 (0.055%)		0.210	< .0001	24 (0.069%)	0.332	< .0001	21 (0.061%)	0.283	< .0001
≥ 60	172 (0.064%)	0.234	< 0.001	184 (0.052%)	0.253	< .0001	220 (0.069%)		0.249	< .0001	129 (0.050%)	0.241	< .0001	127 (0.047%)	0.216	< .0001
10–19	28 (0.066%)	Ref		37 (0.040%)	Ref		28 (0.055%)		Ref		24 (0.069%)	Ref		21 (0.061%)	Ref	
≥ 60	172 (0.033%)	0.911	0.6492	184 (0.052%)	1.249	0.217	220 (0.069%)		1.185	0.398	129 (0.050%)	0.726	0.150	127 (0.047%)	0.763	0.250
10–50	687 (0.197%)	Ref		843 (0.143%)	Ref		797 (0.206%)		Ref		507 (0.181%)	Ref		560 (0.204%)	Ref	
≥ 50	345 (0.083%)	0.400	< .0001	390 (0.068%)	0.457	< .0001	439 (0.090%)		0.419	< .0001	248 (0.064%)	0.355	< .0001	287 (0.071%)	0.347	< .0001

*OR* odds ratio, *CI* confidence interval.

## Discussion

There was a stable prevalence of BOT over time in this nationwide study in South Korea. The median age at diagnosis, prevalence peaked age, type of surgery, and surgical approach were not different during the study periods. Age has consistently correlated with BOT occurrence, although SES was not associated with BOT, except in 2014 (low SES was higher risk of BOT, p < 0.05). Women in their 40 s were at higher risk of BOT than were those 10–19 and ≥ 60 years. The prevalence of BOT was not different in women aged 10–19 years compared to that in women > 60 years old. In contrast, BOT was more prevalent in women aged < 50 years than it was in those ≥ 50 years.

According to our study, BOT is most prevalent in South Korea in women in their 40 s, with a median age of approximately 44 years. BOT was more prevalent in women < 50 years old than it was in those ≥ 50 years of age. In a population-based study in Denmark, the majority of BOT cases were diagnosed in women aged 40–59 years with a median age of 53 years, which should increase the incidence of serous borderline ovarian tumors among women 60–69 years old^4^. Based on data from the Swedish Cancer Registry, the median age at diagnosis with BOT was 61.6 years^3^. In a population-based study of BOT in California, in contrast, the median age at diagnosis was 46 years, which was consistent with our results^6^. Interestingly, in two studies conducted in Asia, the median age at diagnosis was younger than what we found: 39.3 in Taipai^7^ and 38 in Singapore^8^. As such, additional analysis of the risk factors for the difference in prevalent age according to the region is warranted. Nevertheless, careful consideration is needed, given the fact that it was prevalent at relatively young age in Korea.

In ovarian cancer surgery, fertility preservation is only performed for certain patients. In contrast, conservative surgical management of BOT patients is an important consideration for BOT. Conservative surgery is defined as fertility-preserving surgery with complete surgical staging, but with preservation of the uterus and at least part of a unilateral ovary. Since BOT typically occurs in younger women than do other neoplasms, it is important to address fertility preservation. In our study, surgery that preserved the uterus was more prevalent than was surgery with hysterectomy. This discrepancy was also related to the average age of diagnosis of BOT (40–45 years). Nevertheless, the problem with cancer progress should not be overlooked compared to the issue of fertility preservation. Fertility preservation was found to be an independent prognostic factor for recurrence in a large cohort study as was a residual tumor with incomplete staging^9^. However, the risk of malignant recurrent disease was higher in older patients than it was in younger patients^10^. In patients with BOT, the recurrence rates and overall survival were not necessarily correlated because most recurrences were borderline lesions that were cured surgically^11,12^. Overall, the recurrence rates were higher in patients < 40 years old than they were in patients ≥ 40 years. However, there was no difference in overall survival between these two groups, because most of the fertility-sparing surgery was performed in younger patients. Most recurrences occurred in the remaining ovarian tissue with no malignant transformation^13^. Nevertheless, we should consider the risk of invasive recurrence in this setting.

One previous study showed that the postoperative prognosis was poorer in older patients than it was in younger patients with recurrent invasive carcinomas^10^. Biological factors might affect the BOT recurrence. The progression from BOT to an invasive tumor was proven to be associated with a mutation in KRAS and BRAF genes^14–16^. Overall, these genetic mutations are more prevalent with age^17^. Nevertheless, the recurrence often appears as high-grade rather than low-grade, which warrants further investigation.

The extent of staging surgery and the surgical approach in BOT remain very controversial, although surgical removal is the cornerstone of management. Careful inspection of the peritoneum during surgical staging is crucial in patients who are likely to develop recurrent BOT or invasive carcinoma. Although BOT has a good prognosis overall, recurrence free survival and overall survival were associated with proper surgical staging (which includes exploration of the entire abdominal cavity, omentectomy, removal of all peritoneal implants, and multiple peritoneal biopsies)^18^. In this study, approximately 20% of patients underwent surgical staging. Unfortunately, complete surgical staging was only performed in approximately one-third of BOT patients in a previous report; however, 12.5% of BOTs were stage II–IV disease^19^. Prior evidence suggests that the recurrence rate of BOT is 8.0–9.9% after incomplete staging, compared to 1.7% after complete staging^20,21^. The nature of the implant was found to be the most important prognostic factor in advanced stages of BOT. A previous study of 80 advanced stage BOT patients showed that the only factor predicting recurrent invasive disease was the presence of an invasive implant. The 5-year recurrence rates is 31% with an invasive implant, versus only 2% without an invasive implant^22^.

The socioeconomic status affects the incidence of several cancers, including gynecologic cancers^23^. The incidence rates of BOTs were highest among women with low educational level in Denmark, which was consistent with our results in 2014^4^. Women with lower socioeconomic status generally share the following characteristics: higher parity, younger age at first delivery, and higher prevalence of smoking and obesity. Mucinous BOT is more prevalent in smokers, while serous BOT was associated with obesity^24,25^. The prevalence of BOT was not associated with SES between 2015 and 2018, which may be due to historical factors. Therefore, further investigation of the association between socioeconomic status and the histologic type of BOT is warranted.

Our study has several limitations. First, we could not assess the patients’ history of ovarian neoplasm(s) or surgical history because HIRA-NIS is not a longitudinal dataset. For the same reason, we did not evaluate risk factors beyond age and SES. A second limitation is that we did not have detailed surgical information because of limitations in the information that was available from operative codes. For example, oophorectomy and cystectomy could not be distinguished. Furthermore, we could not distinguish unilateral salpingo-oophorectomy from bilateral salpingo-oophorectomy. Third, the pathologic findings could not evaluated, because our data were obtained from diagnostic codes. Therefore, histology-specific trends were not examined. Despite these limitations, our study is meaningful in that it is the first to analyze the recent prevalence and management status of BOT in South Korea.

This nationwide study of BOT in South Korea found that the prevalence trends of BOT stabilized between 2014 and 2018. The BOT prevalence was significantly correlated to patients’ age, and occurred most common in the 5th decade of life. Although fertility-sparing surgery is important for younger patients, proper surgical staging is no less essential for cancer prognosis. Further studies are needed to investigate the time trend of age-specific BOT occurrence and its potential risk factors.

## Methods

South Korea provides medical insurance services from the National Health Insurance Corporation (NHIC) to almost of all people living in the country. The NHIC provides medical insurance services for most diseases. Therefore, it also contains medical information, including age, sex, income, diagnosis, and management^26^. The Health Insurance Review and Assessment Service (HIRA) is an organization that assesses the medical expenses paid by medical institutions. The HIRA determines whether the cost is appropriate and proposes that the corporation pay for it. Therefore, the HIRA and NHIC database contains information on all claims for approximately 50 million Koreans. The HIRA data are only available to a few researchers, because these data have accessibility limitations given their large size and complexity.

The HIRA-National Inpatient Sample (HIRA-NIS) is annual sample data based on a stratified randomized sampling method provided by the HIRA. A probabilistic weighted sample extraction method was utilized to build the HIRA-NIS^26^. The HIRA-NIS extracts data from 13% of inpatients and 1% of outpatients during a one-year period. The HIRA-NIS is sampled annually. We selected data from the Korean Health Insurance Review and Assessment Service-National Inpatient Sample datasets between 2014 and 2018.

The Korean Standard Classification of Disease (7th edition) was used to obtain data which was adapted from the International Statistical Classification of Disease (10^th^ edition). Women with BOT were defined by the presence of the diagnostic code for BOT (D39.1). The Health Insurance Medical Care Expenses were used to access the procedure codes. Women who underwent surgery with BOT were defined by the presence of both diagnostic codes and surgery codes: (Adnexectomy, unilateral R4331, Adnexectomy, bilateral R4332, Transposition of ovary R4413, Extirpation of Adnexal Tumor-Benign R4421, Extirpation of Adnexal Tumor-Malignant, simple R4423, Extirpation of Adnexal Tumor-Malignant, simple (with hysterectomy) R4427, Extirpation of Adnexal Tumor-Malignant, Radical R4424, Extirpation of Adnexal Tumor-Malignant, radical (with hysterectomy) R4428, Ovarian Wedge Resection R4430). Fertility-sparing surgery was defined as whether hysterectomy was performed. Surgical staging surgery was defined as surgical codes including radical surgery (R4424 and R4428).

In order to investigate the surgical approach, laparoscopic surgery was defined as laparoscopic surgical material claim codes (N0031001), while laparotomy was defined by the absence of these codes. A single port approach was defined by wound retractor material claim codes for single port assess: M2055009, M2055011, M2055013, M2055014, M2055023, M2055024, M2055034, M2055037, M2055039, M2055050, M2055051, M2055060, M2055071, M2055073, M2055081, M2055083, M2055085, M2055091, M2055108, M2055138, M2055177, M2055212. In contrast, conventional laparoscopy was defined as an absence of these codes.

Sub-analysis was performed according to age group. Low socioeconomic status is defined by the use of a non-general insurance code, such those used for recipients of livelihood programs and household individuals.

### Statistical analysis

The χ^2^ test was used to compare continuous variables during the study periods. The year and age were designated as independent variables. The diagnosis of BOT and operation codes were designated as dependent variables, for which odds ratios (OR) and 95% confidence intervals (CI) were calculated with logistic regression. A P value < 0.05 was considered statistically significant. All statistical tests were two-tailed. Weighted analyses were used to compare the means of continuous variables. The weighted Pearson’s chi-square test was used for statistical analyses of categorical variables.

### Ethics

The study protocol was approved by the Institutional Review Board (Kangwon National University Hospital Institutional Review Board (IRB) (KNUH-2020-05-004-001)) in accordance with the Declaration of Helsinki. The Institutional Review Board also approved that requirement for informed consent was waived because we used only de-identified information for participants collected from the HIRA.

## Supplementary Information

Supplementary Information.
