# Supplementary material for: Nationwide population-based study of prevalence and trend of borderline ovarian tumors in the Republic of Korea
Source: Sci Rep. 2021 May 27;11:11158. doi: 10.1038/s41598-021-90757-8 (PMC8160013; doi:10.1038/s41598-021-90757-8)
Supplement: Supplementary file 1 — Supplementary Information. [file 41598_2021_90757_MOESM1_ESM.docx]

**Prevalence and trend of borderline ovarian tumors in the Republic of Korea from 2014–2018: a nationwide population-based study**

Yung-Taek Ouh^1^, Dongwoo Kang^2^, Hoseob Kim^2^, Jae Kwan Lee^3^, Jin Hwa Hong^3*^

^1^Department of Obstetrics and Gynecology, School of Medicine, Kangwon National University, Kangwon

^2^Data Science Team, Hanmi Pharmaceutical. Co. Ltd., Seoul, Korea

^3^Department of Obstetrics and Gynecology, Korea University Guro Hospital, College of Medicine, Seoul, Korea.

Supplementary Table 1. Stratified 5-year patient age ranges of BOT patients in 2017 and 2018.

|  | 2017 | | 2018 | |
| --- | --- | --- | --- | --- |
| Age | Number | % | Number | % |
| 5-9 | 2 | 0.26% | - | 0.00% |
| 10-14 | 2 | 0.26% | 5 | 0.59% |
| 15-19 | 22 | 2.91% | 16 | 1.89% |
| 20-24 | 48 | 6.34% | 56 | 6.61% |
| 25-29 | 73 | 9.64% | 103 | 12.16% |
| 30-34 | 94 | 12.42% | 90 | 10.63% |
| 35-39 | 70 | 9.25% | 88 | 10.39% |
| 40-44 | 90 | 11.89% | 92 | 10.86% |
| 45-49 | 108 | 14.27% | 110 | 12.99% |
| 50-54 | 68 | 8.98% | 90 | 10.63% |
| 55-59 | 51 | 6.74% | 70 | 8.26% |
| 60-64 | 37 | 4.89% | 41 | 4.84% |
| 65-69 | 27 | 3.57% | 31 | 3.66% |
| 70-74 | 23 | 3.04% | 17 | 2.01% |
| 75≤ | 42 | 5.55% | 38 | 4.49% |
